# The Effect of a DC Magnetic Field on the AC Magnetic Properties of Oleic Acid-Coated Fe_3_O_4_ Nanoparticles

**DOI:** 10.3390/ma16124246

**Published:** 2023-06-08

**Authors:** Michele Modestino, Armando Galluzzi, Maria Sarno, Massimiliano Polichetti

**Affiliations:** 1 Department of Physics “E.R. Caianiello”, University of Salerno, Via Giovanni Paolo II 132, 84084 Fisciano, SA, Italy; mmodestino@unisa.it (M.M.); agalluzzi@unisa.it (A.G.); msarno@unisa.it (M.S.); 2 CNR-SPIN Salerno, Via Giovanni Paolo II 132, 84084 Fisciano, SA, Italy; 3 NANO_MATES Research Centre, University of Salerno, Via Giovanni Paolo II 132, 84084 Fisciano, SA, Italy

**Keywords:** Fe_3_O_4_ magnetic nanoparticles, superparamagnetism, super spin glass, Mydosh parameter, blocking temperature, AC susceptibility, DC field effect

## Abstract

The AC magnetic properties of a sample of Fe_3_O_4_ nanoparticles coated with oleic acid have been investigated with the help of AC susceptibility measurements. In particular, several DC magnetic fields have been superimposed on the AC field, and their effect on the magnetic response of the sample has been analysed. The results show the presence of a double peak structure in the imaginary component of the complex AC susceptibility measured as a function of the temperature. A preliminary evaluation of the Mydosh parameter for both peaks gives the information that each one of them is associated with a different state of interaction between nanoparticles. The two peaks evolve both in amplitude and position when the intensity of the DC field is changed. The field dependence of the peak position shows two different trends, and it is possible to study them in the framework of the currently existing theoretical models. In particular, a model of non-interacting magnetic nanoparticles has been used to describe the behaviour of the peak at lower temperatures, whereas the behaviour of the peak at higher temperatures has been analysed in the framework of a spin-glass-like model. The proposed analysis technique can be useful for the characterisation of magnetic nanoparticles used in several types of applications, such as biomedical and magnetic fluids.

## 1. Introduction

### 1.1. Superparamagnetism

The properties of magnetic nanoparticles (MNPs) have been widely studied in the last years, and many different techniques are used today for their characterisation [1]. This is due to the large interest in this type of material in terms of its use for biomedical applications [2,3], including drug delivery [4] or hyperthermia cancer therapy [5,6], but also for applications such as data [7] energy storage [8] and for magnetic fluid preparation, which is a colloidal solution of magnetic nanoparticles coated with a stabiliser dispersed in a carrier liquid [9,10,11]. The possibility of MNPs showing a superparamagnetic state above the so-called “blocking temperature” (T_B_) is the main reason for their versatility. This state is similar to the paramagnetic one because of the absence of spontaneous magnetisation, but the prefix “super-” refers to a higher response to the applied magnetic field than the classical paramagnetic state [12,13]. These nanoparticles are obtained from materials that show common magnetic properties in bulk form (either ferromagnetic, ferrimagnetic or antiferromagnetic). With their lower dimensions, MNPs lose the domain structure of bulk magnetic materials and become single-domain magnets. Similar to bulk materials’ domains, in single-domain nanoparticles, there is a magnetic order of the spin. Due to this order, the magnetism of the nanoparticles can be described by a total magnetisation vector whose orientation depends on the competition between the anisotropic effects, interaction with external fields and thermal energy [1,13]. In particular, the orientation of the magnetisation can flip between the equilibrium positions described by the form of the energy. The probability to have these flips (or equivalently, the time necessary to observe a flip) depends on the energy competition. In the absence of an external field, the competition is dominated only by the anisotropic energy (proportional to volume) and the thermal energy (proportional to the temperature). By lowering the dimensions of the particles, the relaxation time of the magnetisation decreases enough to become proportional to the measuring time and to observe the system in a paramagnetic-like state [1]. This is the superparamagnetic state.

### 1.2. Presence of Double T_B_ in Literature

For the reasons described in the last paragraph, the dimensions of MNPs have a great effect on T_B_. In fact, Micha et al. [14] have shown how the dimension distribution of magnetic nanoparticles is linked to the distribution of the T_B_ of the sample, but the dimension is not the only characteristic that can affect it. Moreover, the intensity of the dipole–dipole interaction among nanoparticles [15] or the presence of a cover [16] can affect the blocking temperature. The dipole–dipole interaction can also introduce magnetic frustration in a system of MNPs, leading to a spin-glass-like phase. This behaviour is often referred to as a super-spin-glass system [17], and it has been widely observed and reported in the literature [17,18,19]. This wide range of phenomena can have an additional degree of complexity given by the possibility to observe two different blocking temperatures for the same sample. This can be seen both in direct current (DC) and alternating current (AC) magnetic measurements, but in DCs, the presence of two blocking temperatures can be noted only after elaboration of the data [20,21] using the method proposed by Micha et al. [14]. On the other hand, the presence of two T_B_ can be directly observed via measuring in AC fields. This is reported by Goya et al. [22] where the described phenomenon is due to the presence of nanoparticles with a different dimension distribution in the same sample but also by Urian et al. [21] in the case of interacting nanoparticles. In addition to this, Echevarria-Bonet et al. [23] have shown how in a sample of antiferromagnetic nanoparticles, it is possible to observe a double-phase transition due to the coexistence of super antiferromagnetic and super-spin-glass phases.

### 1.3. Analysis of a System Having Double T_B_

Due to the possibility of effect combinations in MNPs samples, it is important to develop analysis methodologies in order to associate the correct phase transition to the presence of double blocking temperatures. A possibility is to use the effect of a DC superimposed magnetic field (H_DC_) on AC susceptibility measurements. The idea is that depending on the type of transition that characterises the nanoparticles, the DC field produces a different influence on the blocking temperature [24,25,26,27]. In particular, for a super-spin-glass system, the presence of a DC field produces a variation of the freezing temperature (related to the super-spin-glass transition) [28] that is different from the one usually expected for the blocking temperature in a sample of non-interacting MNPs [29,30]. The results obtained with this approach can be compared with the evaluation of the Mydosh parameter [31]. In a similar way to the effect of a superimposed DC field, the effect of the AC field frequency on the blocking temperature is different for non-interacting nanoparticles with respect to a super-spin-glass system. In particular, by evaluating the variation of the blocking temperature in a decade of frequencies, it is possible to empirically separate three ranges for the value of the Mydosh parameter in the case of non-interacting, clusterised and super-spin-glass nanoparticle systems [17,28]. In this work, a MNPs sample has been studied showing the presence of a double peak in the imaginary part of AC susceptibility. The evaluation of the Mydosh parameter has been used to have a preliminary estimation of the behaviour related to the two peaks. After this, the effect of the DC field on the peaks’ temperature has been shown, and a method to analyse the nature of the interaction among the nanoparticles has been provided.

## 2. Materials and Methods

### 2.1. Sample Preparation

In this work, a sample of magnetite nanoparticles covered with oleic acid (Fe_3_O_4_-OA) was analysed. The nanoparticles were synthesised via thermo decomposition of organo-metallic precursor. This method has the advantage of producing nanoparticles with controlled size, high crystallinity and well-defined shape [32]. The precursor used for the synthesis of the nanoparticles characterised in this work was the acetylacetonate of iron, Fe(acac)3. A total of 3 mmol of this precursor was mixed with 20 mL of benzyl ether, 10 mmol of 1,2-hexadecanediol and 12 mmol of oleic acid (OA). The result of this mixture was magnetically stirred during thermal treatment. It was warmed up to 473 K, remaining at this temperature for 120 min, and then it was warmed up to 558 K for 60 min. After this process, the result was washed by centrifugation for 30 min of duration at 7500 rpm firstly in ethanol and then in an equal volume mixture of hexane and ethanol [20]. 

### 2.2. Characterisation Techniques

Sarno et al. [33] also report the characterisation via FT-IR that proves the bonding between magnetite and oleic acid and thermogravimetry measurements that estimate the percent in weight of oleic acid as 20%. Moreover, TEM analyses were performed to estimate the mean diameter of nanoparticles as 6.9 nm with standard deviation of 1.7 nm. It is important to note that TEM measurements showed a monomodal distribution of the MNPs’ dimensions. To study the magnetic properties of the sample, a PPMS (Physical Property Measurement System) by Quantum Design equipped with ACSM insert was used to perform AC susceptibility measurements. The ACSM insert measures different harmonics of the magnetic signal. In particular, the system used in this work was able to acquire ten different harmonics. Due to the AC nature of the measured signal, for each harmonic, a real and imaginary component are distinguished. In this work, we have focused our attention on the first harmonic signal, both real and imaginary components. Before each measurement, the sample was warmed up to a temperature of 300 K and then cooled down to 10 K without any applied field (“Zero Field Cooling” conditions—ZFC). After this, the sample was warmed from 10 K up to 300 K at a rate of 0.5 K/min while measured with an AC-applied field with an amplitude of 12 Oe and a frequency of 107 Hz and 9693 Hz. After performing a measurement without DC field superimposed to the AC one, 4 different DC field values (100 Oe, 400 Oe, 700 Oe and 1000 Oe) were used. To properly control the DC magnetic field and avoid eventual spurious effects due to the presence of a residual magnetic field trapped in the PPMS superconducting magnet [34], this was reduced below 1 Oe using demagnetisation cycles. This procedure starts by setting the field at the value of 20,000 Oe. After this, the field was set at the value of 1 Oe, but this value was approached by oscillated mode. In this mode, the field oscillates through positive and negative values, reducing progressively the amplitude of the oscillations to approach the set field [35].

## 3. Results and Discussion

**Figure 1:**
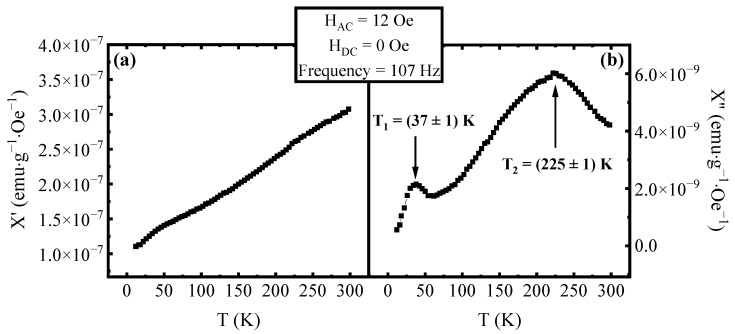
Temperature dependence of (**a**) the real part χ′(T) and (**b**) the imaginary part χ″(T) of the AC magnetic susceptibility at an AC field frequency of 107 Hz, an AC field amplitude of 12 Oe and without a superimposed DC field. In χ″(T) curve, the presence of two peaks in the curve is highlighted, and the temperatures associated with them are reported.

**Figure 2:**
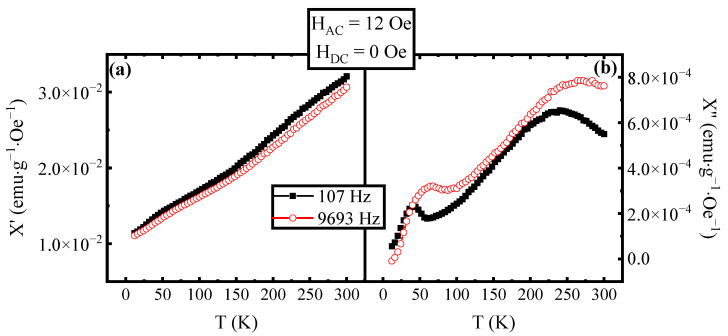
(**a**) χ′(T) curves and (**b**) χ″(T) curves at an AC field amplitude of 12 Oe without a superimposed DC field and at AC field frequency of 107 Hz and 9693 Hz. The curve measured at the frequency of 107 Hz is plotted with filled black squares, while the curve measured at 1077 Hz is plotted with empty red circles.

The evaluation of the real (χ′) and imaginary (χ″) components of AC magnetic susceptibility as a function of temperature is useful to investigate the presence of superparamagnetic transition in MNPs. In fact, as described in the literature [1], the peak in χ′(T) is associated with the T_B_ of the MNPs with the largest dimension. On the other hand, the peak in the χ″(T) curve is associated with the mean value of the blocking temperatures of the MNPs [25]. In Figure 1, the χ′(T) and χ″(T) curves are reported as measured on our sample in the absence of a DC magnetic field. As it is possible to see the real component χ′(T) does not show the presence of a peak, indicating that the nanoparticles with the largest size transition to the superparamagnetic state at temperatures higher than 300 K. On the other hand, χ″(T) shows two peaks, the first at the temperature T_1_ = 37 ± 1 K and the second at the temperature T_2_ = 225 ± 1 K. As reported by Urian et al. [21], the presence of two peaks in χ″(T) can be explained in terms of a dipolar–dipolar interaction among the MNPs. To confirm the presence of interaction among nanoparticles, a measurement at different frequencies was performed. In Figure 2, the χ′(T) curves (Figure 2a) and the χ″(T) curves (Figure 2b) measured at the frequencies of 107 Hz and 9693 Hz of the AC field are plotted. As can be seen, the effect of an increasing frequency is the increase of both T_1_ and T_2_ as expected from the literature [1,25]. Using the value of the peaks’ temperatures in Figure 2b for both frequencies, it is possible to evaluate the Mydosh parameter [31] following the definition:

Φ = ΔT_p_/(T_Lf_ Δlog(f)) (1)

where f is the frequency of the AC magnetic field, T_P_ is the peak’s temperature and T_Lf_ is the peak’s temperature at low frequency. Typically, the value obtained for Φ is in the range of 0.05–0.18 for a super-spin-glass system and in the range of 0.3–0.5 for a system of non-interacting nanoparticles [28]. 

**Figure 3:**
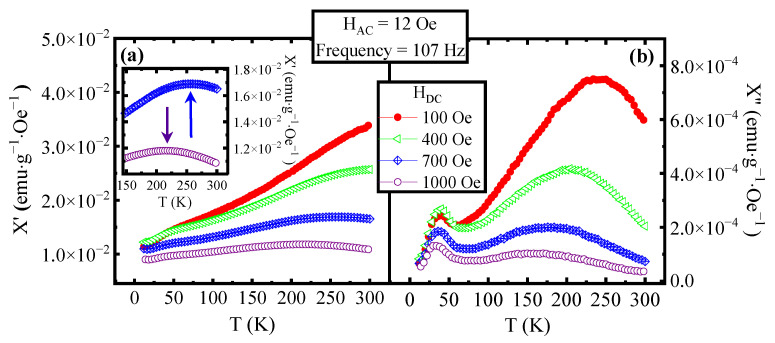
(**a**) χ′(T) curves and (**b**) χ″(T) curves at an AC field frequency of 107 Hz, an AC field amplitude of 12 Oe and with different DC fields superimposed. The curve measured at 100 Oe is plotted with filled red circles, the one at 400 Oe with open green triangles, the one at 700 Oe with crossed blue rhombus and the one at 1000 Oe with open purple circles. In the inset, the χ′(T) curves measured with a DC field of 700 Oe and 1000 Oe are magnified in the high-temperature region where the peaks in the curves, indicated by the arrows, are visible.

The intermediate region is associated with clustered magnetic nanoparticles. In our case, starting from the data in Figure 2, we have obtained Φ_1_~0.33 from T_1_ at 107 Hz, and at 9693 Hz, Φ_2_~0.073 from T_2_ at the same two frequencies. These results suggest that the peak at a lower temperature is associated with a non-interacting MNP behaviour, and the second is associated with a super-spin-glass behaviour. To strengthen these results, a study with different applied DC fields superimposed on the AC one was performed. In Figure 3, the χ′(T) curves (Figure 3a) and the χ″(T) curves (Figure 3b) at different values of the DC fields are reported. It can be noted that for DC applied fields ≥ 700 Oe, a peak appears in the χ′(T) curve (see inset in Figure 1a), confirming the superparamagnetic transition for the largest nanoparticles and consequently for the entire sample. The temperature shift of the peak is in agreement with the theory that predicts a decrease in the blocking temperature with the increase of a DC field applied to the MNPs [29].

Observing Figure 3b, it can be noted that the position of the peak at low temperature (T_1_) shifts just slightly by increasing the DC field, while the position of the peak at high temperatures (T_2_) varies much more.

**Figure 4:**
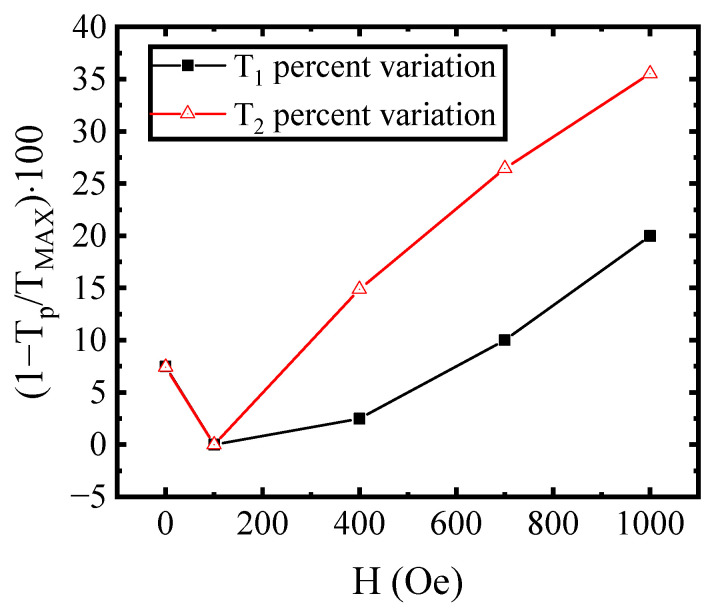
Percentage variation for T1 and T2 compared to their maximum value as function of DC applied field. The T1 percentage variation is plotted with filled black squares, while the T2 percentage variation is plotted with open red triangle.

This can be better seen by plotting the percentage difference between the maximum value of T_1_ (equivalently T_2_), corresponding to the curve at the DC field of 100 Oe, and the other values of T_1_ (respectively T_2_) at different DC fields, as reported in Figure 4. It can be noted that, except for changing the DC field from 0 Oe to 100 Oe, the T_2_ percentage variation with a field is larger than the T_1_ one. 

**Figure 5:**
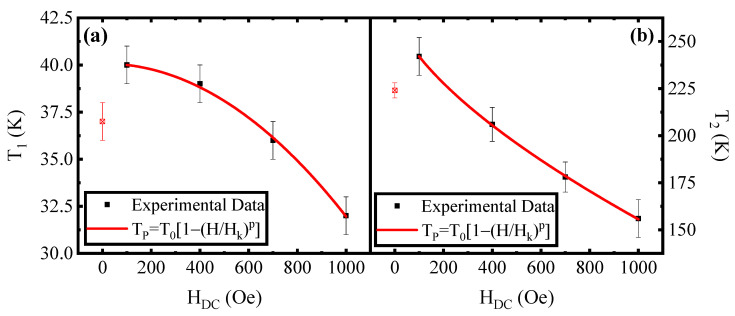
(**a**) T_1_ (H_DC_) and (**b**) T_2_(H_DC_) behaviours (with associated error bars). The (red) continuous lines are the best fit obtained by using Equation (4) for both the sets of data. The points at low field that do not match with the fit are marked with an open square symbol (in red).

This can suggest that the physical mechanisms associated with the two peaks are different. In order to confirm this hypothesis, a quantitative analysis of the T_1_ and T_2_ values as a function of the applied DC field was performed. The results are shown in Figure 5 where the different DC field dependences of the peaks’ temperatures are evidenced in the range from 100 Oe to 1000 Oe. As shown by Mamiya and Nakatani [36], for non-interacting nanoparticles, the temperature associated with the peak of χ″(T) at different DC fields follows the relation:

T_B_ = T_B0_ [1 − (H/H_an_)^2^] (2)

where T_B0_ is blocking temperature at zero fields, and H_an_ is the anisotropy field; that is the field for which the anisotropy energy becomes lower than the magnetic energy.

On the other hand, in the literature, the existence of a super-spin-glass behaviour for MNPs is also reported [28,37,38]. In that case, the field dependence of the peak temperature (T_SSG_) of χ″(T) follows the relation:

T_SSG_ = T_f_ [1 − (H/H_0_)^2/3^] (3)

where T_f_ is the freezing temperature of the system without a magnetic field, and H_0_ is the theoretical transition field for a spin-glass state at zero temperature.

For these reasons, our experimentally detected dependences of both T_1_ and T_2_ on the magnetic field (see Figure 5) have been fitted with the more general relation:

T_P_ = T_0_ [1 − (H/H_k_)^p^] (4)

which takes into account both Equation (2) and Equation (3) and where T_P_ is the equivalent of T_B_ and T_SSG_, H_an_ and H_0_ have been substituted by H_k_, T_0_ represents T_B0_ and T_f_, while the exponent p is considered a fit parameter. To perform the fit, the parameter T_0_ has been derived in terms of H_k_ and p by fixing the point T_1_ (or alternatively T_2_) at H_DC_ = 100 Oe as T_100_.

T_0_ = T_100_/[1 − (100/H_k_)^p^] (5)

In this way, the fit is reduced to have two parameters, and the value of T_0_ can be derived from Equation (5) after the evaluation of H_k_ and p. This has been performed because, in our case, both T_1_ and T_2_ have an opposite (increasing) field behaviour in the range from 0 Oe to 100 Oe with respect to the higher fields’ region, as already mentioned. This has a consequence that the points at 0 Oe are not in agreement with the model described by Equation (5), and the parameter T_0_ cannot be initialised by T_1_ and T_2_ at H_DC_ = 0 Oe. In Figure 5, the red line represents the result of the fitting procedure. In Table 1, the parameters obtained by the fit are reported for both the field dependences T_1_(H_DC_) and T_2_(H_DC_). The values obtained for the parameter p indicate that the behaviour of T_1_ (H_DC_) is well-described by Equation (2), whereas that of T_2_ (H_DC_) follows Equation (3).

These results suggest that Peak 1 at low temperatures is associated with non-interacting nanoparticles, and Peak 2 is associated with nanoparticles with a spin-glass-like behaviour. Both behaviours can be explained by considering a single distribution of magnetic nanoparticles with different states of aggregation. It is possible that in our sample, some MNPs are non- or weakly interacting (Peak 1), and others have a strong interaction, showing a super-spin-glass behaviour (Peak 2). This possibility is also supported by a similar behaviour observed in the literature [39,40,41] and could be better investigated in the future by studying the third harmonic signal of the sample [40,42]. All the results obtained by the fit proposed in Figure 5 are valid in non-zero DC fields. On the contrary, as mentioned, the data taken for H_DC_ = 0 Oe do not match this description since the experimental values of the peak temperatures associated with this field are lower than what should be expected from the fit procedure (T_0_ in Table 1) for both curves. This non-monotonic behaviour was already observed for samples of MNPs by Aslani et al. [43], Sappey et al. [44] and Luo et al. [45]. One possible interpretation of this behaviour can be the formation/decay of aggregates caused by the dipole–dipole interaction [10,11]. Zheng et al. [46] have described it starting by considering nanoparticles with a large dimension distribution. In the case of the sample analysed in this work, as previously observed in Figure 1a, the blocking temperature associated with the biggest nanoparticles is higher than 300 K for H_DC_ = 0 T. On the other hand, the temperatures associated with the peaks of the χ″(T) curves are (37 ± 1) K and (225 ± 1) K for H_DC_ = 0 T. These greatly different values can be associated with a wide dimension distribution, as deduced from Equation (5) that defines the blocking temperature [47].

T_B_ = KV/(k_B_ ln(1/(f_m_/τ_0_))) (6)

in which K is the coefficient of anisotropy, V is the volume of nanoparticles, k_B_ is the Boltzmann constant, f_m_ is the measurement frequency and τ_0_ is the characteristic relaxation time of the nanoparticles. The direct proportionality between the blocking temperature and the dimension of the nanoparticles suggests that a large difference in the blocking temperature values corresponds to a large difference in the dimensions of the nanoparticles, making Zheng’s description for the non-monotonic trend of T_B_ (H_DC_) also applicable to the results reported in this work. It is important to note that the H^2/3^ dependence of T_B_ is predicted as possible also for non-interacting MNPs, and for this reason, the occurrence of this kind of dependence is not enough to distinguish a super-spin-glass system from a non-interacting system. However, the H^2/3^ dependence of T_B_ is reported for DC fields close to H_an_ [29,48]. In our case, we have estimated H_k_ for the T_1_ (H_DC_) dependence as (2200 ± 100) Oe (Table 1), and, in agreement with theory [29], we have found T_1_ ∝ H^2^ for H < H_k_. The H^2/3^ law found for T_2_ (H_DC_) dependence cannot be associated with non-interacting nanoparticles of the same family as those associated with the peak at lower temperature T_1_ because the DC fields used in this work are lower than the H_k_ estimated for the peak at temperature T_1_. It could be also possible that the peak at T_2_ is associated with another family of MNPs with an H_k_ low enough to obtain H^2/3^ dependence of T_2_. However, this hypothesis can also be rejected because H_k_ is proportional to the anisotropic energy KV [29,48,49]. If the nanoparticles associated with the peak at T_2_ have an anisotropic energy lower than the one of the nanoparticles associated with the peak at T_1_, according to Equation (5), T_1_ has to be higher than T_2_ due to the proportionality between T_B_ and KV. This hypothesis is clearly in disagreement with the experimental data, so it is more reasonable that the peak at T_2_ is associated with MNPs that show a super-spin-glass behaviour. Following this idea, the value of H_k_ has been evaluated for the peak at higher temperatures as H_k_ = (3900 ± 200) Oe, which is in accordance with the value obtained for systems of the same typology [28,37].

## 4. Conclusions

The dependence of the blocking temperature as a function of the applied DC field has been studied in order to analyse the interaction among nanoparticles in a sample of oleic acid-coated Fe_3_O_4_ characterised by a double peak structure in χ″(T). The values calculated for the Mydosh’s parameter are Φ_1_~0.33 for the peak at a lower temperature and Φ_2_~0.076 for the peak at a higher temperature. According to the literature, the first is in the range associated with non-interacting nanoparticle behaviour, whereas the second is associated with a spin-glass-like system. The first result obtained via the evaluation of the Mydosh parameter has been investigated from the analysis of AC susceptibility under different DC fields. The DC field dependence of the temperature associated with the two peaks was studied by using a general power law model. The obtained results have shown that the peaks can be ascribed to the presence of two different magnetic mechanisms. In particular, the DC field dependence of the peak at lower temperatures well fits the behaviour expected in the case of non-interacting nanoparticles. On the other hand, a spin-glass-like model result is more suitable to analyse the behaviour of the higher temperature peak. This also made it possible to estimate the value for the anisotropic field from the T_1_ (H_DC_) behaviour and for the theoretical transition field from the T_2_ (H_DC_) behaviour. Finally, the used models are unable to correctly predict the blocking temperature value at a zero DC field due to the wide distribution of the MNPs’ dimensions in our sample. In general, for samples with double T_B_, the proposed study can be useful in combination with TEM analysis to investigate the effects due to dipole–dipole interactions and the distribution of dimensions.

## Figures and Tables

**Table 1:** Fit parameters for T_1_(H_DC_) and T_2_(H_DC_). The value presented for parameter T_0_ is not obtained directly by fit but by the evaluation of Equation (5) in which, for H_k_ and p, the values reported in this table are used.

Fit Parameter	T_1_ (H_DC_) Fitting	T_2_ (H_DC_) Fitting
T_0_	(40.07 ± 0.02) K	(267 ± 2) K
H_k_	(2200 ± 100) Oe	(3900 ± 200) Oe
p	2.03 ± 0.11	0.65 ± 0.03
Adjust R-Square	0.9978	0.99965

## Data Availability

The data sets that support the findings in this study are available from the corresponding author upon reasonable request.
